# Springboard to an academic career—A national medical student research program

**DOI:** 10.1371/journal.pone.0195527

**Published:** 2018-04-30

**Authors:** Geir W. Jacobsen, Helge Ræder, Marianne H. Stien, Ludvig A. Munthe, Vegard Skogen

**Affiliations:** 1 Norwegian University of Science and Technology – NTNU, Faculty of Medicine and Health Sciences, Trondheim, Norway; 2 University of Bergen, Faculty of Medicine, Bergen, Norway; 3 KG Jebsen Centre for B cell Malignancies, University of Oslo, Faculty of Medicine, Oslo, Norway; 4 UiT Arctic University of Norway, Faculty of Health Sciences, Tromsø, Norway; Karolinska Institutet, SWEDEN

## Abstract

Over the last decades there has been a decline in the recruitment of medical students into academia in all medical fields. Concurrently, medical research has increasingly included other disciplines in multidisciplinary convergence, introducing an unmet recruitment gap and requirement for medical researchers. To counteract the trend and recruit students to academic medicine, a national intercalated Medical Student Research Program (MSRP) was established in Norway in 2002. A preliminary evaluation in 2009 suggested that the MSRP had resulted in recruitment, but could not conclude on a lasting effect beyond graduation in a study that did not include any controls. These results led us to hypothesize that the MSRP could increase the number of PhD degrees and attract medical students towards academic medicine. Adopting a case cohort design, we here report that the intercalated MSRP had a significant impact of the throughput of physician-scientists to PhD, by increasing the rate of PhD completion 10-fold (p<0.001). Moreover, almost twice as many MSRP physicians reported an academic aspiration (49% vs 22%, p<0.001). Results suggested that an MSRP-like approach could efficiently address the unmet recruitment gap and strengthen the medical disciplines in medical research.

## Introduction

During the 1990s there was a steady decrease in the proportion of medical candidates in Norway who pursued an academic career and completed a doctoral degree [1]. This mirrored international trends and caused concern as the decline involved all medical fields, including basic, clinical, paraclinical, and public health research [2–5].

A number of initiatives have since been launched to counteract the trend. These initiatives have mostly focused on medical undergraduates and have been classified as either extracurricular [3,6,7] or intracurricular activities, and in the latter case, as mandatory [8–10] or elective/intercalated programs [11–14]. Moreover, similar initiatives have been reported for medical graduates in academic pediatrics and psychiatry [15,16]. More recently, a review underscored the need for evidence from longitudinal follow-up that included the residency period and early clinical careers to confirm that undergraduate medical student research programs truly facilitated the development of scholars [17]. Other reports have focused on how to identify the preferred qualities of future physician-scientists [18] and to fuel interest and retain enthusiasm for research among medical undergraduates [19]. Yet others have raised ethical concerns in case the quality of undergraduate research involving humans does not merit publication [20].

Following an initiative taken by the Norwegian Medical Faculties, the Research Council of Norway, and the Norwegian Department of Education and Science, a Medical Student Research Program (MSRP) was conceived and funded. The program was organized in concert and launched simultaneously at all four faculties in 2002. The MSRP was well received by the stakeholders, students, and Faculty academics and administrations. In 2009, a detailed evaluation of the first five years (2002–6) was reported [12]. Although the analysis only covered the short start-up period, it was suggested that the program led to an overall increase in the recruitment of medical candidates to biomedical and health related research. Nevertheless, the first evaluation allowed no conclusions on the MSRP program on dissertation frequency and academic career aspirations [12]. Although promising, the study design did not allow controlled analysis of the effects of the MSRP.

To follow up this study and to answer the call for controlled longitudinal study [17] and to conclude, we designed a case cohort study where we put forward the hypothesis that the MSRP increased the rate of PhD degrees and that the program increased the aspirations for scientific careers. Thus, we conducted a national controlled questionnaire study among all medical candidates who have completed the MSRP since 2002 through the academic year 2013/14.

Further, we also aimed to characterize the MSRP cohort in terms of time to completion of the PhD, the areas of research, publication merits, vocational training and clinical track records, as well as current professional academic status and impact on specialist training.

## Materials and methods

We designed a case cohort study and conducted a questionnaire survey among MDs from the four Medical Faculties in Norway who had completed the MSRP. We covered the whole period from the program was launched in 2002 and through the academic year 2013/2014. The MSRP MDs were compared with a control group that consisted of two individually age and sex matched class peers from the year of admission to their respective medical faculties, but who had not applied for admission to the program. Both groups received the same questionnaire and an invitation letter that explained the objectives of the study. The procedure aimed to achieve study cohort homogeneity and avoid potential bias caused by curricular and organizational differences between medical faculties and changes that may have occurred within separate faculties during the observation period.

The questionnaire had 29 items and explored demographic characteristics such as the students’ sex, year of birth, ethnic background, name of medical school, and year of entry and graduation [S1 Questionnaire]. Questions about career development regarded information about internship and vocational/specialty training, current main occupation and position, type of institution (university, regional or local hospital) vs. non-hospital affiliation, and ambition to pursue an academic career. Scientific characteristics included numbers of published scientific articles, conference presentations, research abroad, a completed PhD or aims/ambitions to obtain one, and area and type of research.

We identified as cases every medical candidate who had been enrolled in the program from the inception in 2002 and had obtained a medical degree (MD) between 2006 and spring 2014. They were matched individually as 1 case per 2 controls as described above. Potential control candidates were identified by the four Medical Faculty administrations. We thereby identified 374 case (MSRP) and 696 control MDs.

### Procedures

The data collection took place between 19 January and 11 February 2015. The questionnaires were designed in SurveyXact^®^, and a link to the survey was distributed by email to three groups; MDs who completed their MSRP before 2007 [12], MDs who completed their MSRP after 2007, and a control group, consisting of 2 controls per case, matched by gender, age and year of entry to medical school.

The questionnaires were distributed by email 19 January 2015 with a first and second reminder on 3 and 11 February, respectively. The survey was closed 19 February 2015.

### Statistics

Data [S1 Dataset] were analyzed using STATA, Version 14 (College Station, Texas, USA). Univariate and bivariate methods were employed for continuous and Fisher’s exact statistics for categorical variables. The two tailed Mann Whitney U-test was calculated by GraphPad Prism v.6.0 (GraphPad Software La Jolla California USA, www.graphpad.com). P-values < .05 were considered statistically significant.

### Ethics

The study was approved by NSD—Norwegian Centre for Research Data, which is a subsidiary of the Norwegian Data Inspectorate.

## Results

**Fig 1:**
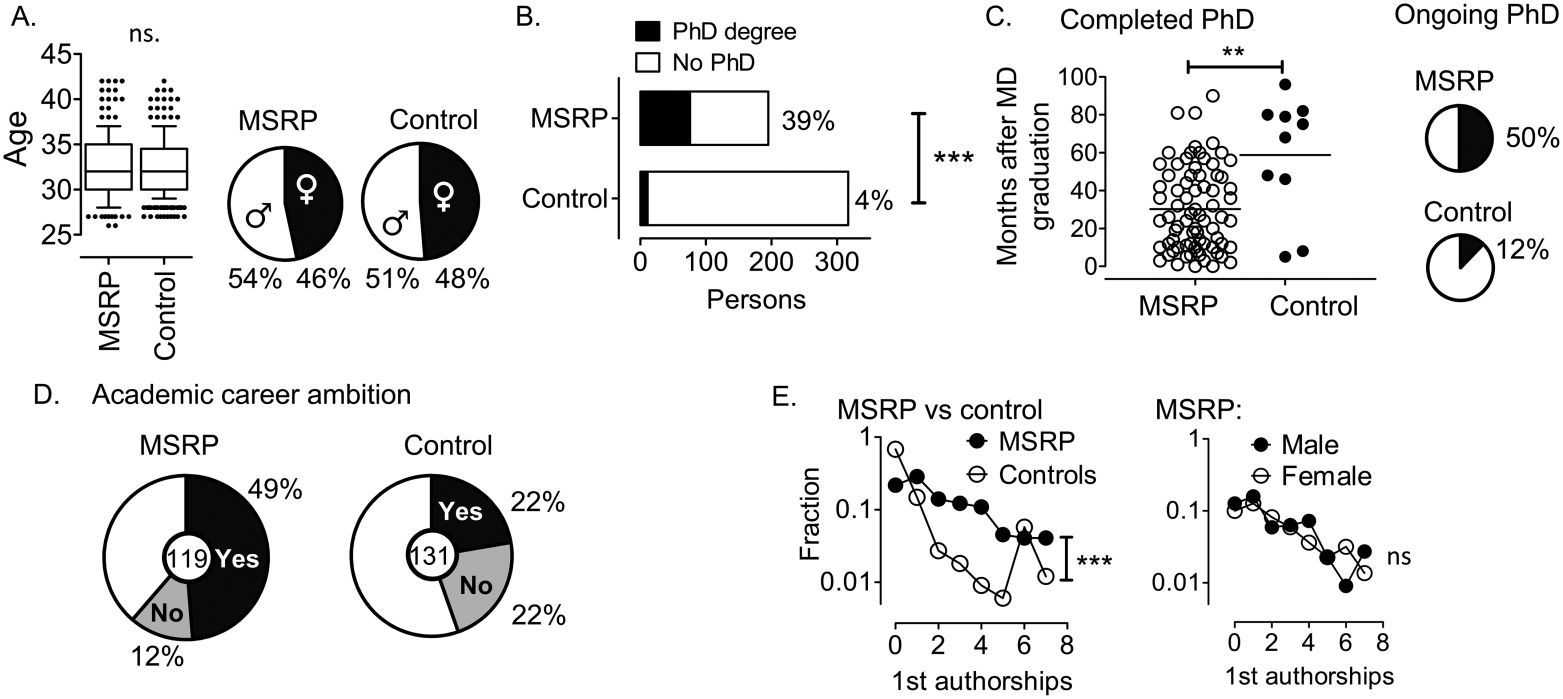
Analysis of the MSRP and control cohorts. A. Left: Age of MSRP-graduates compared to control, dot plot with box whiskers and 10–90% confidence interval with outliers is shown. Right: Gender distribution in MSRP and control. B. Stacked histograms showing number of PhD graduates (black filed histogram) and MDs without PhD (white histogram), MSRP vs. control. Fisher’s exact test, p&lt;0.0001. C. Left: PhD graduates are shown, scatter dot plot shows the number of months from completing MD education to dissertation, MSRP vs. controls, P&lt;0.008, Mann Whitney U test, two tailed. Right: Pie charts showing MSRP graduates and controls and fraction that were enrolled in PhD program, Fisher’s exact, P&lt;0.0001. D. Academic career ambitions, pie chart shows fraction of “yes” (black segments), “no” (gray) or “do not know” (white) responses. E. Left: Fraction of MSRP graduates or control that have 0, 1, 2, 3, 4, 5, 6, or 7 first authorships, P&lt;0.0001, Mann Whitney U test, two tailed. Right: MSRP graduates subdivided into male and female, P = 0.75, Mann Whitney U test, two tailed test.

Before reporting the responses to answer our hypothesis question (see below), we here first define the case and control populations. We received a total of 538 completed questionnaires from 221 MSRP graduate cases (61% response rate) and 317 controls (45% response rate). The MSRP cases were on average aged 32.6 and the controls were 33.5 years old (Fig 1A), (two tailed Mann-Whitney U test, P = 0.99). The gender distribution was similar in the groups (Fig 1A). Further, comparable proportions worked at a university level hospital, either as residents, in vocational/specialty training or as a consultant in a university hospital, or in general practice (Table 1).

**Table 1:** Reported career characteristics among Norwegian MDs who had been enrolled in the Medical Student Research Program (MSRP) or had followed the regular undergraduate medical curriculum, the 2006–14 cohort.

Career characteristic	MSRP (n = 221)	Control—(n = 317)
Hospital residents	73 (33%)	148 (47%)
Vocational/Specialty training	100 (of 124 replies, 81%)	224 (of 253 replies, 88%)
Consultants	6 (2.7%)	13 (4.1%)
University position	2 (0,1%)	1 (0.003%)
General practitioners	16 (7.2%)	24 (7,6%)

We next focused on our hypothesis that the MSRP program increased the rate of PhD graduates. Of the 221 MSRP cases, 195 returned answers as to whether they had graduated for the PhD degree. For the control group, 301 of 371 submitted an answer. We found a 10 times increased rate of completed PhDs in the MSRP group (39% vs 4% (Fig 1B), Fisher’s exact, p<0.0001). Moreover, these graduates defended their dissertations in about half the time after MD graduation, a result that was significantly different from the controls (Fig 1C). In the remaining populations that had not completed a PhD, 50% of the MSRP graduates were in process of conducting PhD research, compared to 12% in the control group (Fisher’s exact, p<0.0001, Fig 1C). The data therefore showed a significant increase in PhDs among MSRP graduates.

We further followed up by characterizing the MSRP group. Half (49%) of them reported that they had an academic career ambition, while the corresponding proportion among the controls was 22% (Fig 1D). In terms of scientific production, a significant higher fraction of MSRP graduates had published one or more paper as first author, as second author, or as co-author (Fig 1E) and data not shown. Further, we found no differences in the track record of male and female MSRP graduates in terms of publication rate (Fig 1E and data not shown). Also, more than twice as many MSRP graduates (9.8%) than controls (4.4%) reported that they had spent at least 3 months abroad as research affiliates. However, the mean time from graduation as an MD to specialization was 10 years (SE 0.15) for MSRP candidates compared to 8.5 years among controls, which was a significant increase (P<0.001).

## Discussion

We found that the MSRP significantly increased the PhD completion rate about 10-fold and that the program also doubled the academic aspirations of MSRP graduates. As for other parameters, the time before completion of the PhD thesis was significantly reduced while the publication track was also significantly higher. On the other hand, specialization took on average 1.5 years longer for former MSRP students.

The response rate (61%) among the cases was acceptable, as was the technical quality of their responses. Our results were convincing since the observed differences in proportion and speed of completed PhDs or proportion of ongoing ones, number of scientific publications, academic track aspirations, as well as time needed to become a specialist were more distinct than expected.

We were unable to identify other studies that had conducted a similar controlled follow-up of the impact of a structured academic program for medical undergraduates. Hence, we seem to be among the first to respond to a call for longer follow-up of undergraduate student researchers [17]. This literature review showed that undergraduate medical research efforts were “typically not characterized longitudinally through later stages of the participants’ careers”. Moreover, the authors found it hard to “illuminate participants’ future research engagement and productivity”. In the current study, we could draw the conclusion that a focused program such as the MSRP made a very significant impact on scientific activities, career choices, and aspirations. In extension, it is likely that the program facilitates the development of scholars.

A paper by Smith et al [16] describes a program initiated by The American Pediatric Society and Society for Pediatric Research in 1991. The program supported medical students with interests in research and pediatrics to conduct research at institutions other than their respective medical schools. Ten years later, participants had published more actively than nonparticipant applicants, while male and female applicants had published equally. By 2008, 36% of the program participants were in pediatrics and 29% in academic pediatrics, respectively [16].

A Swedish questionnaire study was recently published [9]. The authors reported a follow-up of close to 400 medical students who had been enrolled in a 20-week mandatory research project 2 years before interview. One third of the students reported that they had co-authored one or more scientific publications, and or had given oral presentations. A similar proportion had a future academic career as part of their professional plans. However, PhD ambitions seemed to decrease with age, but were equally distributed between sexes. On the other hand, the younger respondents were more reluctant to do research in the future, possibly due to concerns on financial prospects [21]. It was also apparent that students interacted professionally with their supervisors even after their completed course-work. Like others [19,22], the authors suggested that further studies should focus on the role of the supervisor as a longer-term mentor.

Two other European papers support our findings. First, a Swiss survey showed that a considerable portion of former MD/PhD graduates had an impressive publication record and that half of those who completed their PhD thesis early on, had chosen an academic career [23]. However, the study had no control group. A recent Dutch paper concluded that participation in a scientific pre-university program (SPUP) increased the number of medical students who wished to pursue a career as clinician-scientists [24]. The effect was most evident when they were compared to an unmatched group of non-SPUP medical students, but less so when the comparison groups were matched on students who excelled in some other way after admission to medical school. Thus, the authors admitted that their crude outcomes might have been influenced by self-selection.

There were several limitations, first the lower (45%) response rate among the controls. That was in part anticipated and was one reason why we invited two controls per case. Thus, we failed to make contact with as many as 52 (= 7.4%) controls. However, the average age and sex distribution were similar between the respondents. Had it not been for the observed differences between the groups, we might have faced an underpowered study.

The questionnaire method is prone to misclassification in either direction and possibly differentially for cases and controls. Yet, information about clinical and academic merits was readily available, for instance via PubMed.

There is of course a possibility that the MSRP students had increased academic career ambitions prior to applying to the program. Thus, we may surmise that they would attain their academic results regardless of the program and with the same efficiency. Even so, their overall career will no doubt benefit from an extended number of years as academics.

It may be argued that intercalated MD/PhD programs already have resulted in more academic physicians with a PhD and academic productivity [25–27] and that our results do not add much to the field. We hold that a direct comparison between our intercalated MSRP and North American MD/PhD programs is not straightforward. For instance, a Norwegian MD comprises the completion of a full time 6 years curriculum which is at odds with a typical North American template of 4 (BSc) + 4 (MD) years. Our academic frame requires a postgraduate program of another 3 full years before an MD may attain a top (PhD) academic level. The current study demonstrates that the MSRP had halved this time. Second, our MSRP program recruits interested applicants once or twice a year, these participants are followed more closely as an embedded sub-cohort of 10–12 scientific novices among their student peers. Admittance is based on a self-composed scientific protocol, a written statement from a designated senior academic, and an in-depth interview in front of a panel that also includes a more senior MSRP student. Third, a national week-end scientific conference is organized annually; this is led by current MSRP students and alumni, and rotates among the universities. These events are supported academically and financially by the institutions, are formative, and add momentum to *l’esprit de corps*.

The current study will be followed up with further analysis of the MSRP cohort in terms of further longitudinal analysis of the research productivity and attainment of top academic positions.

## Conclusions

Our results demonstrate the efficiency of targeted programs in undergraduate medical education; it is likely that such actions will be required in several educational settings across Europe. Further follow-up will be required to demonstrate prolonged efficiency.

## Supporting information

S1 Dataset Selected information collected from Norwegian MSRP students and controls, the 2006–14 cohort. (XLSX)

S1 Questionnaire English translation of the questionnaire used by Norwegian MSRP students and controls, the 2006–14 cohort. (DOCX)
