# Supplementary material for: Springboard to an academic career—A national medical student research program
Source: PLoS One. 2018 Apr 30;13(4):e0195527. doi: 10.1371/journal.pone.0195527 (PMC5927424; doi:10.1371/journal.pone.0195527)
Supplement: S1 Questionnaire — (DOCX) [file pone.0195527.s002.docx]

## **Supplementary Information, Geir W. Jacobsen et al**

This document is a translation of the questionnaire that was presented to the cases and controls in the study.

## **Evaluation - Norwegian Medical Student Research Program (MSRP) 2014-15**

## **QUESTIONNAIRE**

Thank you for taking the time to respond to this survey. Your answers are important to us.

**1. Where did you study medicine?**

(1) ❑ UiB University of Bergen

(2) ❑ UiO University of Oslo

(3) ❑ NTNU Norwegian University of Science and Technology - Trondheim

(4) ❑ UiT Arctic University of Norway - University of Tromsø

**2a. Do you have medical school diploma with MSRP?**

(1) ❑ Yes

(2) ❑ No

(3) ❑ No, I started out, but quit the MSRP along the way

**2b. Why did you quit the MSRP?**(1) ❑ Lost interest in the project

(2) ❑ Didn’t find “my place” in the research group

(3) ❑ Problematic relationship with supervisor

(4) ❑ Personal reasons

**3. What is your gender?**

(1) ❑ Male

(2) ❑ Female

**4. What year were you born?**
____

**5. Do you have an immigrant background?**

(1) ❑ No

(2) ❑ Yes, western

(3) ❑ Yes, non-western

**6. What year did you enter medical school?**
____

 **7. How many semesters of your regular medical school curriculum did you complete before you were accepted to the MSRP? (ie. when you received your first research scholarship)**(1) ❑ 1 semester

(2) ❑ 2 semesters

(3) ❑ 3 semesters

(4) ❑ 4 semesters

(5) ❑ 5 semesters

(6) ❑ 6 semesters

(7) ❑ 7 semesters

(8) ❑ 8 semesters

(9) ❑ 9 semesters

(10) ❑ 10 semesters

8. How long did you stay with the MSRP (number of semesters)?

((1) ❑ 1 semester

(2) ❑ 2 semesters

(3) ❑ 3 semesters

(4) ❑ 4 semesters

(5) ❑ 5 semesters

(6) ❑ 6 semesters

(7) ❑ 7 semesters

(8) ❑ 8 semesters

(9) ❑ 9 semesters

(10) ❑ 10 semesters

9. How will you classify your research?

(1) ❑ Epidemiology / Community Health Research

(2) ❑ Clinical research

(3) ❑ Laboratory / preclinical / paraclinical research

(4) ❑ Other type of research __________

10. Did you ever consider quitting the MSRP?

(1) ❑ No

(2) ❑ Yes

11. What was the reason why you considered to quit?

(1) ❑ Lost interest in the project

(2) ❑ Didn’t find “my place” in the research group

(3) ❑ Problematic relationship with supervisor

(4) ❑ Personal reasons

12. How many hours of supervision did you receive as an MSRP student during the last year? (from your main supervisor, co-supervisors and other competent staff in the research group)

(1) ❑ Less than 20 hours

(2) ❑ 20-40 hours

(3) ❑ 41-80 hours

(4) ❑ More than 80 hours

13. How often did you receive supervision?

(1) ❑ At least once a week

(2) ❑ Every two weeks

(3) ❑ Regularly, but less than every two weeks

(4) ❑ Occasional

(5) ❑ No supervision

14. To what extent were you satisfied with the supervision?

(1) ❑ To a very large extent

(2) ❑ To a large extent

(3) ❑ To some extent

(4) ❑ To a small extent

(5) ❑ To a very small extent

15. Did you feel you needed more supervision than you received?

(1) ❑ Yes

(2) ❑ No

(3) ❑ Don’t know

16. How would you characterize the expectations your supervisor had for you?

(1) ❑ Very high expectations

(2) ❑ High expectations

(3) ❑ Medium expectations

(4) ❑ Low expectations

(5) ❑ Very low expectations

17. To what extent do you think the expectations of your supervisor were consistent with your own?

(1) ❑ To a very large extent

(2) ❑ To a large extent

(3) ❑ To some extent

(4) ❑ To a small extent

(5) ❑ To a very small extent

18. Did you publish any scientific or popular scientific articles as an MSRP student?

(1) ❑ Yes

(2) ❑ No

(3) ❑ Submitted for review (number) __________

19a. Authorship of published articles

|  | 0 | 1 | 2 | 3 | 4 | 5 |
| --- | --- | --- | --- | --- | --- | --- |
| Articles as 1. author | (6) ❑ | (1) ❑ | (2) ❑ | (3) ❑ | (4) ❑ | (5) ❑ |
| Articles as 2. uthor | (6) ❑ | (1) ❑ | (2) ❑ | (3) ❑ | (4) ❑ | (5) ❑ |
| Articles as co-author | (6) ❑ | (1) ❑ | (2) ❑ | (3) ❑ | (4) ❑ | (5) ❑ |

19b. Authorship - PubMed (PMID No.) 8 digits

| Articles as 1.author: | ______________________________ |
| --- | --- |
| Articles as 2.author: | ______________________________ |
| Articles as co-author: | ______________________________ |

20. How many presentations (oral or poster) did you give at national/regional congresses as an MSRP student (total)?

(1) ❑ 0

(2) ❑ 1

(3) ❑ 2

(4) ❑ More than 2

21. How many presentations (oral or poster) did you give at international congresses as an MSRP student (total)?

(1) ❑ 0

(2) ❑ 1

(3) ❑ 2

(4) ❑ More than 2

22. **Did you spend a research stay abroad as an MSRP student?**

(1) ❑ No

(2) ❑ Yes, number of weeks (total) ________

23. **How satisfied were you with the following as an MSRP stundent?**

|  | Very satisfied | Quite satisfied | Neither/Nor | Quite dissatisfied | Very dissatisfied |
| --- | --- | --- | --- | --- | --- |
| The research group | (1) ❑ | (2) ❑ | (3) ❑ | (4) ❑ | (5) ❑ |
| Your project | (1) ❑ | (2) ❑ | (3) ❑ | (4) ❑ | (5) ❑ |
| Your own efforts | (1) ❑ | (2) ❑ | (3) ❑ | (4) ❑ | (5) ❑ |
| Progress of the project | (1) ❑ | (2) ❑ | (3) ❑ | (4) ❑ | (5) ❑ |
| Service and support from the administration | (1) ❑ | (2) ❑ | (3) ❑ | (4) ❑ | (5) ❑ |
| Organized workplace | (1) ❑ | (2) ❑ | (3) ❑ | (4) ❑ | (5) ❑ |

24. To what extent do you think your experiences as an MSRP student were beneficial for you as a regular medical student?

(1) ❑ To a very large extent

(2) ❑ To a large extent

(3) ❑ To some extent

(4) ❑ To a small extent

(5) ❑ To a very small extent

25. Overall, how satisfied were you as an MSRP student?

(1) ❑ Very satisfied

(2) ❑ Quite satisfied

(3) ❑ Neither satisfied nor dissatisfied

(4) ❑ Quite dissatisfied

(5) ❑ Very dissatisfied

26. When did you receive your medical diploma?

(22) ❑ Spring-06

(23) ❑ Fall-06

(24) ❑ Spring-07

(25) ❑ Fall-07

(26) ❑ Spring-08

(27) ❑ Fall-08

(11) ❑ Spring-09

(12) ❑ Fall-09

(13) ❑ Spring-10

(14) ❑ Fall-10

(15) ❑ Spring-11

(16) ❑ Fall-11

(17) ❑ Spring-12

(18) ❑ Fall-12

(19) ❑ Spring-13

(20) ❑ Fall-13

(21) ❑ Spring-14

27. Have you any published scientific or popular science articles after you completed the MSRP?

(1) ❑ Yes

(2) ❑ No

(3) ❑ Submitted for review (number) __________

**28a. Authorship of published articles - Number**

|  | 0 | 1 | 2 | 3 | 4 | 5 |
| --- | --- | --- | --- | --- | --- | --- |
| Articles as 1. author | (6) ❑ | (1) ❑ | (2) ❑ | (3) ❑ | (4) ❑ | (5) ❑ |
| Articles as 2. author | (6) ❑ | (1) ❑ | (2) ❑ | (3) ❑ | (4) ❑ | (5) ❑ |
| Articles as co-author | (6) ❑ | (1) ❑ | (2) ❑ | (3) ❑ | (4) ❑ | (5) ❑ |

**28b. Authorship - PubMed (PMID No. 8 digits). Use punctuation marks (,) if you have more than 1 publication)**Articles as 1st author: ______________________________
Articles as 2nd author: ______________________________
Articles as co-author: ______________________________


**29. How many presentations (oral or poster) have you given at national/regional congresses, total?**(1) ❑ 0

(2) ❑ 1-5

(3) ❑ 6-10

(4) ❑ More than 10

**30. How many presentations (oral or poster) have you given had at international congresses, total?**(1) ❑ 0

(2) ❑ 1-5

(3) ❑ 6-10

(4) ❑ More than 10

**31. Have you had research stays abroad > 3 months after the MSRP?**

(1) ❑ Yes, total number of months _____

(2) ❑ No

**32. Have you finished a PhD degree?**

(1) ❑ Yes

(2) ❑ No

**33. How many months after your graduation from medical school were you admitted to the PhD program?
____**
**34. How many months after finishing medical school did you defend your PhD thesis?
____**
**35. Are you currently in a PhD program?**(1) ❑ Yes

(2) ❑ No

**36. Would you like to complete a PhD degree?**

(1) ❑ Yes

(2) ❑ No

(3) ❑ Don’t know

**37. On what project do you want to take a PhD degree?**

(1) ❑ Continuation of the MSRP project

(2) ❑ Another project

**38. At which university?**

(1) ❑ UiB

(2) ❑ UiO

(3) ❑ NTNU

(4) ❑ UiT

(5) ❑ Another university in Norway

(6) ❑ Another university abroad

**39. How is / was your PhD project funded?**
(1) ❑ Regional Health Authority

(2) ❑ University

(3) ❑ Norwegian Research Council

(4) ❑ The Norwegian Cancer Society

(6) ❑ Norwegian Institute of Public Health

(7) ❑ “Health and Rehabilitation Foundation”

(8) ❑ Other funding

**40. How would you classify your research?**

(1) ❑ Epidemiology / Community Health Research

(2) ❑ Clinical research

(3) ❑ Laboratory / preclinical / paraclinical research

(4) ❑ Other type of research __________

**41. Have you finished your internship?**

(1) ❑ Yes, finished

(2) ❑ Yes, on my way

(3) ❑ No

**42. Are you currently doing your specialization?**
(1) ❑ Yes

(2) ❑ No


**43. What year did you start your specialization?**____

**44. What specialty do you aim for?**

(1) ❑ General practice

(2) ❑ Anesthesiology

(3) ❑ Occupational medicine

(4) ❑ Child and adolescent psychiatry

(5) ❑ Pediatric surgery

(6) ❑ Pediatrics

(7) ❑ Hematology

(8) ❑ Breast and endocrine surgery

(9) ❑ Endocrinology

(10) ❑ Gastroenterology

(11) ❑ Physical and rehabilitation medicine

(12) ❑ Obstetrics and gynecology

(13) ❑ Digestive surgery

(14) ❑ General surgery

(15) ❑ Geriatrics

(16) ❑ Cardiology

(17) ❑ Dermato - venerology

(18) ❑ Immunology and transfusion medicine

(19) ❑ Internal medicine

(20) ❑ Infectious diseases

(21) ❑ Vascular surgery

(22) ❑ Clinical pharmacology

(23) ❑ Clinical neurophysiology

(24) ❑ Respiratory diseases

(25) ❑ Maxillofacial surgery

(26) ❑ Medical biochemistry

(27) ❑ Medical genetics

(28) ❑ Medical microbiology

(29) ❑ Neurosurgery

(30) ❑ Neurology

(31) ❑ Nuclear medicine

(32) ❑ Nephrology

(33) ❑ Oncology

(34) ❑ Orthopedic surgery

(35) ❑ Pathology

(36) ❑ Plastic surgery

(37) ❑ Psychiatry

(38) ❑ Radiology

(39) ❑ Rheumatology

(40) ❑ Community medicine

(41) ❑ Thoracic surgery

(42) ❑ Urology

(43) ❑ Otorhinolaryngology Diseases

(44) ❑ Ophthalmology

**45. Do you have one or more specialization approvals?**(1) ❑ Yes, year of first specialization________

(2) ❑ No

46. What specialty(-ies)(s) do you have today?

(1) ❑ General practice

(2) ❑ Anesthesiology

(3) ❑ Occupational medicine

(4) ❑ Child and adolescent psychiatry

(5) ❑ Pediatric surgery

(6) ❑ Pediatrics

(7) ❑ Hematology

(8) ❑ Breast and endocrine surgery

(9) ❑ Endocrinology

(10) ❑ Gastroenterology

(11) ❑ Physical and rehabilitation medicine

(12) ❑ Obstetrics and gynecology

(13) ❑ Digestive surgery

(14) ❑ General surgery

(15) ❑ Geriatrics

(16) ❑ Cardiology

(17) ❑ Dermato - venerology

(18) ❑ Immunology and transfusion medicine

(19) ❑ Internal medicine

(20) ❑ Infectious diseases

(21) ❑ Vascular surgery

(22) ❑ Clinical pharmacology

(23) ❑ Clinical neurophysiology

(24) ❑ Respiratory diseases

(25) ❑ Maxillofacial surgery

(26) ❑ Medical biochemistry

(27) ❑ Medical genetics

(28) ❑ Medical microbiology

(29) ❑ Neurosurgery

(30) ❑ Neurology

(31) ❑ Nuclear medicine

(32) ❑ Nephrology

(33) ❑ Oncology

(34) ❑ Orthopedic surgery

(35) ❑ Pathology

(36) ❑ Plastic surgery

(37) ❑ Psychiatry

(38) ❑ Radiology

(39) ❑ Rheumatology

(40) ❑ Community medicine

(41) ❑ Thoracic surgery

(42) ❑ Urology

(43) ❑ Otorhinolaryngology Diseases

(44) ❑ Ophthalmology

47. What is your main occupation today?

(14) ❑ Intern

(1) ❑ LIS (Specialist-in-training)

(2) ❑ Consultant

(3) ❑ GP with an official community contract

(15) ❑ GP without an official community contract

(16) ❑ A specialist with an official community contract

(17) ❑ A specialist without an official community contract

(4) ❑ PhD candidate

(5) ❑ Postdoc/researcher

(6) ❑ Permanent academic position (20-100 %) at a university hospital or university/university college

(7) ❑ Unoccupied, temporarily

(8) ❑ Unoccupied, permanently

(9) ❑ Non-medical business

(10) ❑ Main position in the public sector as a doctor

(11) ❑ Main position in the public sector, not as a doctor

(12) ❑ Main position in the private sector as a doctor

(13) ❑ Main position in the private sector, not as a doctor

48. For hospital staff after the internship: Workplace

(1) ❑ Regional Health Authority with university hospital function

(2) ❑ Regional Health Authority with regional hospital function

(3) ❑ Regional Health Authority with local hospital function

(4) ❑ Not applicable

49. Do you have ambitions or wishes for an academic career?

(1) ❑ Yes

(2) ❑ No

(3) ❑ Not sure

Thanks! You have now completed the survey. Your answers are registered.
